# Restoration of pp60^Src^ Re-Establishes Electron Transport Chain Complex I Activity in Pulmonary Hypertensive Endothelial Cells

**DOI:** 10.3390/ijms26083815

**Published:** 2025-04-17

**Authors:** Manivannan Yegambaram, Marissa D. Pokharel, Xutong Sun, Qing Lu, Jamie Soto, Saurabh Aggarwal, Emin Maltepe, Jeffery R. Fineman, Ting Wang, Stephen M. Black

**Affiliations:** 1 Center for Translational Science, Florida International University, 11350 SW Village Parkway, Port St. Lucie, FL 34987-2352, USA; myegamba@fiu.edu (M.Y.); marissa.pokharel@cuanschutz.edu (M.D.P.); sunxutong66@gmail.com (X.S.); qlu@fiu.edu (Q.L.); jamsoto@fiu.edu (J.S.); tinwang@fiu.edu (T.W.); 2 Department of Cellular Biology & Pharmacology, Herbert Wertheim College of Medicine, Florida International University, Miami, FL 33199, USA; saaggarw@fiu.edu; 3 Department of Pediatrics, University of California San Francisco, San Francisco, CA 94143, USA; emin.maltepe@ucsf.edu (E.M.); jeff.fineman@ucsf.edu (J.R.F.); 4 Cardiovascular Research Institute, University of California San Francisco, San Francisco, CA 94143, USA; 5 Department of Environmental Health Sciences, Robert Stempel College of Public Health and Social Work, Florida International University, Miami, FL 33199, USA

**Keywords:** pp60^Src^ kinase, phosphorylation, mitochondria, complex I, pulmonary hypertension, mass spectrometry

## Abstract

It is well-established that mitochondrial dysfunction plays a critical role in the development of pulmonary hypertension (PH). However, the molecular mechanisms and how the individual electron transport complexes (ETC) may be affected are poorly understood. In this study, we identified decreased ETC Complex I activity and assembly and linked these changes to disrupted mitochondrial bioenergetics in pulmonary arterial endothelial cells (PAECs) isolated from a lamb model of PH with increased pulmonary blood flow (Shunt). These derangements were associated with decreased mitochondrial activity of the protein tyrosine kinase, pp60^Src^. Treating Control PAECs with either the Src family kinase inhibitor, PP2, or the siRNA-mediated knockdown of pp60^Src^ was able to recapitulate the adverse effects on ETC Complex I activity and assembly and mitochondrial bioenergetics. Conversely, restoring pp60^Src^ activity in lamb PH PAECs re-established ETC Complex I activity, improved ETC Complex I assembly and enhanced mitochondrial bioenergetics. Phosphoprotein enrichment followed by two-dimensional gel electrophoresis and tandem mass spectrometry was used to identify three ETC Complex I subunits (NDUFS1, NDUFAF5, and NDUFV2) as pp60^Src^ substrates. Finally, we demonstrated that the pY levels of NDUFS1, NDUFAF5, and NDUFV2 are decreased in lamb PH PAECs. Enhancing mitochondrial pp60^Src^ activity could be a therapeutic strategy to reverse PH-related mitochondrial dysfunction.

## 1. Introduction

Mitochondria are essential membrane-bound organelles required for several biological processes in the cell, including energy metabolism [1]. Impaired or altered mitochondrial function decreases energy metabolism and reduces bioenergetics, leading to various metabolic disorders and human pathologies [2,3,4,5,6,7,8,9,10,11], including PH [12,13,14,15]. Prior work has shown that derangements in the electron transport chain are associated with PH development [16,17,18,19,20]. Our prior work has shown that PH-related stimuli decrease Complex I activity in PAECs [21]. However, it is unknown if derangements in ETC Complex I are involved in the development of PH. Thus, this study was designed to investigate the potential role of ETC Complex I disruption in the mitochondrial dysfunction associated with PH development. In addition, as the mechanisms by which the ETC is modulated during PH development are unresolved, we also investigated the potential role of post-translational regulation.

Phosphorylation is a reversible PTM that regulates many cellular processes, including cell survival, proliferation, and death [22,23,24]. Several protein kinases are transported to the mitochondria [25,26,27], where they can regulate the activity of proteins involved in critical mitochondrial processes. These include stabilizing the mitochondrial complexome and controlling the electron transport chain (ETC), TCA cycle, fatty acid metabolism, oxidative stress, and mitochondrial fusion/fission events [28,29]. pp60^Src^ belongs to the Src family of protein tyrosine kinases (SFKs). It is a critical regulator of many physiological functions, including metabolism, growth, proliferation, and angiogenesis [29,30,31,32,33]. pp60^Src^ is localized to many subcellular compartments, including mitochondria, Golgi apparatus, endosomes, and plasma membrane [34,35,36]. pp60^Src^ has been shown to target components of oxidative phosphorylation by modulating ETC activity [30,31,37]. Prior work has demonstrated that mitochondrial pp60^Src^ can modulate ETC Complex I activity [37]. However, the molecular targets have not been resolved, and the potential role of mitochondrial pp60^Src^ (mt-pp60^Src^) in PH development has not been investigated. Therefore, this study also focused on identifying mitochondrial pp60^Src^ (mt-pp60^Src^) kinase targets in ETC Complex I proteins and investigating if pp60^Src^ is critical for preserving Complex I activity.

Our results identified decreased mt-pp60^Src^ levels in PAECs isolated from a lamb model of PH associated with increased pulmonary blood flow (Shunt) [38], which correlated with decreased ETC Complex I activity and assembly and the suppression of mitochondrial bioenergetics. Further, our data show that the inhibition of pp60^Src^ in PAECs using the SRK family inhibitor (PP2) or the siRNA-mediated knockdown of pp60^Src^ recapitulates these adverse effects on ETC Complex I activity and assembly, significantly reducing mitochondrial bioenergetics. Conversely, restoring pp60^Src^ in PH-PAECs increased ETC Complex I activity and assembly and improved mitochondrial bioenergetics. Utilizing mass spectrometry, we identified three mitochondrial ETC Complex I subunits (NDUFS1, NDUFAF5, and NDUFV2) that are substrates of pp60^Src^ and showed that the pY levels of these proteins are attenuated in PH-PAECs. Overall, our data demonstrate that mitochondrially localized pp60^Src^ regulates ETC Complex I activity and mitochondrial bioenergetics through the phosphorylation of NDUFS1, NDUFAF5, and NDUFV2 and suggest that stimulating pY events in Complex I could alleviate the mitochondrial dysfunction associated with PH.

## 2. Results

### 2.1. Pulmonary Arterial Endothelial Cells Isolated from a Lamb Model of PH (Shunt) Have Reduced Mitochondrial Bioenergetics and Decreased ETC Complex I Activity

**Figure 1:**
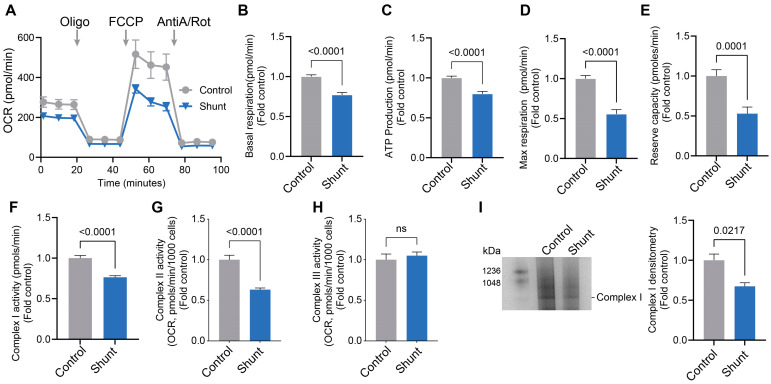
Mitochondrial respiration, Complex I activity and Complex I assembly are attenuated in pulmonary arterial endothelial cells isolated from Shunt lambs. The Agilent Seahorse XF24e analyzer was used to take measurements in PAECs isolated from three twin pairs of Shunt and age-matched Control lambs. Shunt PAECs present with disrupted bioenergetics (**A**) such that basal respiration (**B**), the OCR for ATP synthesis (**C**), and the maximal (**D**) and reserve respiratory capacity (**E**) were significantly decreased. Shunt PAECs show decreased ETC Complex I (**F**) and II (**G**) activities. Complex III activity is unchanged in Shunt PAECs (**H**). Blue native polyacrylamide gel electrophoresis analysis shows decreased Complex I assembly in Shunt PAECs (**I**). Data are presented as means ± SEM. All experiments were performed with PAECs isolated from three independent Shunt lambs and three independent age-matched Control lambs with 10 technical replicates per sample in the Seahorse experiment.

Confirming prior work [39,40,41,42], an OCR analysis using the Seahorse XFe24 extracellular flux analyzer in conjunction with the Mitostress test kit, Shunt PAECs have decreased bioenergetics (Figure 1A) with basal respiration (Figure 1B), the O_2_ consumed for ATP production (Figure 1C), and maximal (Figure 1D) and reserve (Figure 1E) respiratory capacities all being suppressed. Further, the decrease in bioenergetics correlated with attenuated ETC Complex I (Figure 1F) and II (Figure 1G) activities. However, Complex III activity remained unchanged (Figure 1H). Mitochondrial extracts (10 µg) from Control and Shunt PAECs were subjected to blue native polyacrylamide gel electrophoresis (BN-PAGE) to analyze the mitochondrial respiratory Complex I abundance, and reduced Complex I assembly was observed in Shunt PAECs (Figure 1I).

### 2.2. Shunt PAECs Have Reduced Mitochondrial pp60^Src^ Accumulation

**Figure 2:**
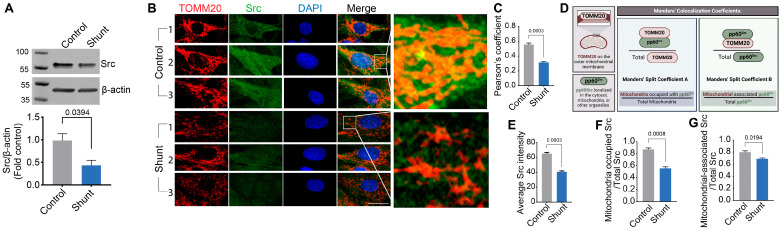
Mitochondrial localization of pp60^Src^ is attenuated in pulmonary arterial endothelial cells isolated from Shunt lambs. Western blot analysis of PAECs isolated from Shunt lambs shows decreased pp60^Src^ protein level compared to PAECs isolated from age-matched Control lambs (**A**). β-actin was used to normalize protein loading. Fluorescent microscopy analysis was carried out using antibodies specific for pp60^Src^ (GREEN) or the mitochondrial protein TOMM20 (RED) in Shunt and Control PAECs. DAPI was used to identify the nucleus (BLUE, (**B**)). The Pearson correlation coefficient shows decreased mitochondrial pp60^Src^ protein levels in Shunt PAECs compared to Control PAECs (**C**). The Manders’ correlation overlap calculation (**D**) confirms decreased total pp60^Src^ levels in Shunt PAECs (**E**). Manders’ correlation coefficient A and B (**D**) identifies decreased pp60^Src^ accumulation in the mitochondria of Shunt PAECs (**F**,**G**). Scale bars: 10 µm. Data are presented as means ± SEM. All experiments were performed with PAECs isolated from three independent Shunt lambs and three independent age-matched Control lambs.

Prior work, including work from our lab, has shown that pp60^Src^ levels and activity are modulated during the development of PH [43,44]. This, in conjunction with work showing that mitochondrially localized pp60^Src^ can affect ETC Complex activity [30,31,37], led us to examine mitochondrial pp60^Src^ levels in Shunt PAECs. Initial Western blot studies indicated that Shunt PAECs have decreased pp60^Src^ protein levels (Figure 2A). Fluorescent microscopy was then used to examine mitochondrial pp60^Src^ protein levels (Figure 2B). Pearson’s coefficient analysis of the fluorescent co-localization images identified decreased mitochondrial pp60^Src^ accumulation in Shunt PAECs (Figure 2C). Manders’ correlation coefficients were used to quantify the degree of colocalization between fluorophores (pp60^Src^ = green; TOMM20 = red/mitochondria) (Figure 2D). The Manders’ coefficient overlap calculation showed decreased pp60^Src^ intensity in Shunt PAECs (Figure 2E). Manders’ split coefficient A (used to estimate the fraction of mitochondria occupying pp60^Src^ to total mitochondria) showed decreased mitochondrial pp60^Src^ accumulation in the Shunt PAECs (Figure 2F). Manders’ split coefficient B (used to estimate the fraction of mitochondria-associated pp60^Src^ to total pp60^Src^) showed decreased mitochondrial pp60^Src^ levels in Shunt PAECs (Figure 2G).

**Figure 3:**
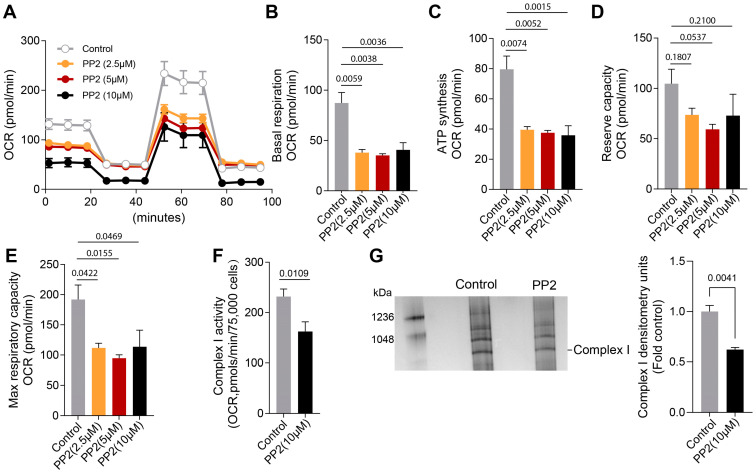
Inhibiting pp60^Src^ alters Complex I activity, Complex I assembly, and mitochondrial respiration in pulmonary arterial endothelial cells. PAECs were treated with increasing concentrations of PP2 (0, 2.5, 5, and 10 µM) for 24h, and the Agilent Seahorse XF24e analyzer was used to measure real-time oxygen consumption rate (OCR). PP2 (2.5, 5, and 10 µM) disrupts the bioenergetic profile for OCR (**A**) such that basal respiration (**B**), the OCR for ATP synthesis (**C**), and the maximum respiratory capacity (**E**) are decreased. The reserve capacity was unchanged (**D**). PP2 (10 µM) attenuates ETC Complex I activity (**F**) in PAECs. Blue native polyacrylamide gel electrophoresis analysis shows that PP2 (10 µM) decreases mitochondrial ETC Complex I assembly in PAECs (**G**). Data are presented as means ± SEM. All experiments were performed with at least three biological replicates.

We next determined if inhibiting pp60^Src^ activity could mimic the effects on Complex I activity we observed in PH-PAECs. To accomplish this, Control PAECs were exposed to increasing concentrations of the pyrazolopyrimidine compound PP2 (0, 2.5-, 5-, and 10-µM), a potent and selective inhibitor of the Src family protein tyrosine kinases [45]. Our data show that the inhibition of pp60^Src^ by PP2 (at various concentrations) disrupted mitochondrial bioenergetics (Figure 3A) such that the basal respiration (Figure 3B), the O_2_ consumed for ATP production (Figure 3C), and the maximal respiratory capacity (Figure 3E) were all decreased. The reserve respiratory capacity was unchanged (Figure 3D). The activity of Complex I was also reduced by PP2 (10 µM) (Figure 3F). Lastly, we investigated if pp60^Src^ inhibition influenced Complex I assembly. PP2-treated PAECs showed decreased Complex I assembly (Figure 3G).

### 2.3. siRNA-Mediated Knockdown of pp60^Src^ Alters Complex I Activity, Complex I Assembly, and Mitochondrial Respiration

**Figure 4:**
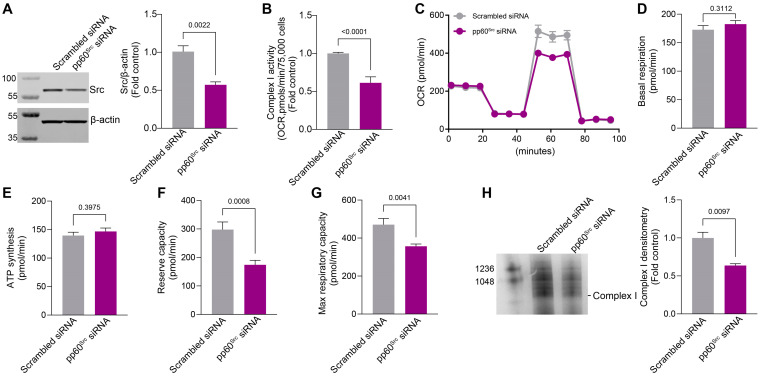
siRNA-mediated knockdown of pp60^Src^ alters Complex I activity, Complex I assembly, and mitochondrial respiration in human pulmonary arterial endothelial cells (HPAECs). HPAECs were transfected with either a scrambled siRNA or an siRNA specific to pp60^Src^ (20 nM) for 48 h. Western blot analysis confirmed decreased pp60^Src^ expression with the specific pp60^Src^ siRNA (**A**). β-actin was used to normalize protein loading. ETC Complex I activity is attenuated by the specific pp60^Src^ siRNA (**B**). HPAECs transfected with the specific pp60^Src^ siRNA show a disrupted bioenergetic profile for OCR (**C**) such that reserve capacity (**F**) and maximum respiratory capacity (**G**) are decreased. Basal respiration (**D**) and the OCR for ATP synthesis (**E**) were unchanged. Blue native polyacrylamide gel electrophoresis analysis shows decreases in the mitochondrial ETC Complex I assembly in the specific pp60^Src^ siRNA transfected HPAECs (**H**). Data are presented as means ± SEM. All experiments were performed with at least three biological replicates.

To confirm our pharmacological data, we investigated whether an siRNA-mediated knockdown of pp60^Src^ would affect Complex I activity, Complex I assembly, and mitochondrial respiration. To accomplish this, HPAECs were transfected with a scrambled siRNA or siRNA specific to pp60^Src^ (20nM) for 48 h. Results from the Western blot analysis confirmed a significant decrease in pp60^Src^ protein levels in the pp60^Src^ siRNA-transfected HPAECs (Figure 4A). Further, a significant reduction in ETC Complex I activity was also observed with the knockdown of pp60^Src^ (Figure 4B). The knockdown of pp60^Src^ also disrupted the bioenergetic profile (Figure 4C) such that reserve- (Figure 4F) and maximum (Figure 4G) respiratory capacities were decreased. Basal respiration (Figure 4D) and the OCR for ATP synthesis (Figure 4E) remained unchanged. The knockdown of pp60^Src^ also decreased mitochondrial ETC Complex I assembly in HPAECs (Figure 4H). Together, these findings link reduced pp60^Src^ mitochondrial localization to the disruption of ETC Complex I activity and attenuated mitochondrial bioenergetics.

### 2.4. Identification of pp60^Src^ Protein Substrates in ETC Complex I

**Figure 5:**
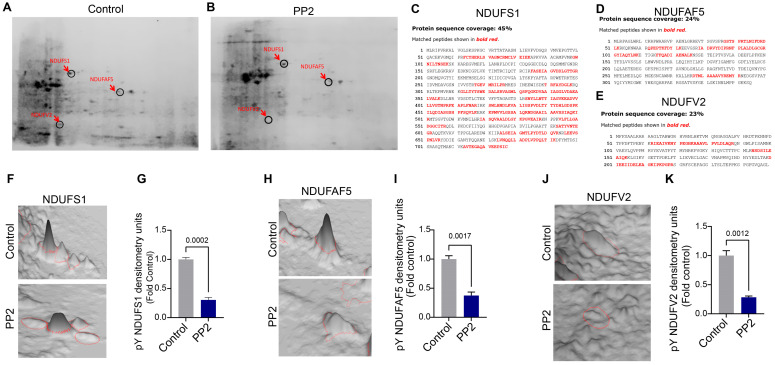
Inhibition of pp60^Src^ modifies mitochondrial protein tyrosine phosphorylation. PAECs were treated with 10 µM PP2 for 24h. Affinity-captured phosphorylated mitochondrial proteins were separated by two-dimensional gel electrophoresis (2D-GE). The protein spots on the 2D gels were visualized using Coomassie staining. PP2 decreased mitochondrial protein tyrosine phosphorylation (**B**) compared to Control PAECs (**A**). MS/MS on the protein spots from the 2D gels identified NDUFS1 (**C**), NDUFAF5 (**D**), and NDUFV2 (**E**) with 45%, 24%, and 23% sequence coverage, respectively. Quantification of protein spots in the 2D gels reveals that PP2 treatment decreased phosphorylation levels in NDUFS1 (**F**,**G**), NDUFAF5 (**H**,**I**), and NDUFV2 (**J**,**K**) compared to the protein spots of respective Controls. Data are presented as means ± SEM. All experiments were performed with three biological replicates.

**Figure 6:**
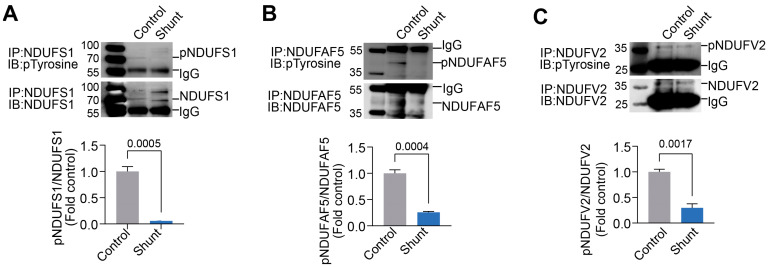
pY levels in NDUFS1, NDUFAF5, and NDUFV5 are decreased in pulmonary arterial endothelial cells isolated from a lamb model of PH. NDUFS1, NDUFAF5, and NDUFV2 proteins were immunocaptured from three twin pairs of Shunt and age-matched Control PAECs and run on one-dimensional electrophoresis gels, immobilized on PVDF membranes, and probed with an anti-phosphotyrosine antibody. Western blot analysis shows that pY levels in NDUFS1 (**A**), NDUFAF5 (**B**), and NDUFV2 (**C**) decreased in Shunt PAECs. Loading was normalized by reprobing gels with the appropriate immunocapture antibody. Data are presented as means ± SEM. All experiments were performed with PAECs isolated from three independent Shunt lambs and three independent age-matched Control lambs.

Using a phosphoprotein enrichment column, we captured mitochondrial pY proteins in Control and PP2-treated PAECs. These proteins were then separated via 2D-PAGE using two orthogonal parameters: isoelectric point (charge) and relative molecular weight. Results from the Coomassie-stained 2D-PAGE gels showed decreased pY protein spot intensity in PP2-treated PAECs (Figure 5A,B). Individual phosphoprotein spots in Control and PP2 treatment were then excised from the Coomassie-stained 2D-PAGE gels, subjected to trypsin/chymotrypsin digestion, and analyzed by mass spectrometry (MS) to identify the protein substrates. This revealed three mitochondrial respiratory Complex I multi-subunits. These were identified by MS as NADH-ubiquinone oxidoreductase 75 kDa subunit (NDUFS1) (Figure 5C), arginine-hydroxylase (NDUFAF5) (Figure 5D), and NADH dehydrogenase [ubiquinone] flavoprotein 2 (NDUFV2) (Figure 5E). Quantification of the NDUFS1 protein spot in the 2D-PAGE gels showed that levels of tyrosine-phosphorylated NDUFS1 (pY-NDUFS1) decreased with PP2 treatment (Figure 5F,G). Similarly, pY-NDUFAF5 levels were reduced with PP2 treatment (Figure 5H,I). Quantification of the pY-NDUFV2 also decreased with PP2 treatment (Figure 5J,K). Next, NDUFS1, NDUFAF5, and NDUFV2 were immunoprecipitated from Control and Shunt PAECs and subjected to Western blot analysis using an anti-pY antibody. The results showed that the pY levels in NDUFS1 (Figure 6A), NDUFAF5 (Figure 6B), and NDUFV2 (Figure 6C) were all attenuated in Shunt PAECs.

### 2.5. Restoration of pp60^Src^ Protein Levels Improves ETC Complex I Activity and Bioenergetics in Shunt PAECs

**Figure 7:**
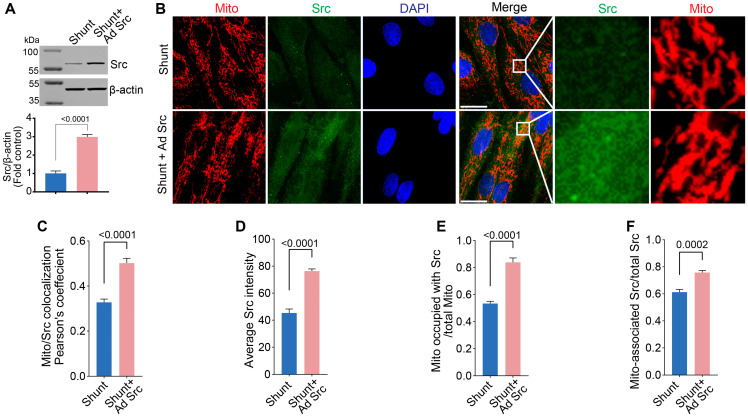
Restoration of pp60^Src^ in Shunt PAECs increases pp60^Src^ localization to the mitochondria. Shunt PAECs were transduced with Ad-CA-Src (MOI = 10) for 48 h. Western blot analysis shows increased pp60^Src^ protein levels in Shunt PAECs upon pp60^Src^ restoration (**A**). β-actin was used to normalize protein loading. Fluorescent microscopy analysis showed increased pp60^Src^ protein levels (GREEN) in Shunt PAECs upon pp60^Src^ restoration (**B**). The Pearson’s correlation coefficient shows increased pp60^Src^ protein levels in CA-Src transduced Shunt PAECs compared to Shunt PAECs (**C**). Manders’ correlation coefficient was used to measure the degree of colocalization of pp60^Src^ and TOMM20 (RED) in the mitochondria. CA-Src transduced Shunt PAECs show increased average pp60^Src^ protein intensity (**D**), increased mitochondria occupied by pp60^Src^ (**E**), and increased mitochondria-associated pp60^Src^ (**F**). Scale bars: 10 µm. Data are presented as means ± SEM. All experiments were performed with at least four biological replicates.

To determine if restoration of pp60^Src^ in Shunt PAECs could positively impact mitochondrial function, we used an adenoviral expression construct to deliver a constitutively active pp60^Src^ mutant (CA-Src) to Shunt PAECs. Using an adenoviral vector (Ad-CA-Src, MOI = 10), pp60^Src^ protein levels were increased by ~3-fold in Shunt PAECs (Figure 7A). Using fluorescent microscopy, we evaluated how this impacted the intensity and mitochondrial localization of pp60^Src^ protein (Figure 7B). Pearson’s coefficient analysis in the fluorescence colocalization study revealed increased mitochondrial pp60^Src^ accumulation in CA-Src-expressing Shunt PAECs (Figure 7C). The colocalization of pp60^Src^ (green) with mitochondrial TOMM20 (red) was then quantified using Manders’ correlation coefficient. The Manders’ overlap coefficient calculation showed increased pp60^Src^ intensity in the cytoplasm of CA-Src-expressing Shunt PAECs (Figure 7D). We also observed an increased number of mitochondria with pp60^Src^ accumulation (Figure 7E) as well as increased pp60^Src^ mitochondrial accumulation (Figure 7F).

**Figure 8:**
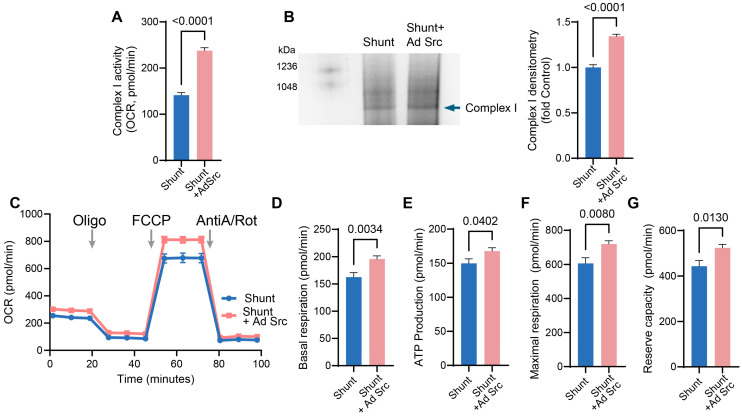
Restoration of pp60^Src^ improves Complex I activity, Complex I assembly, and mitochondrial bioenergetics in pulmonary arterial endothelial cells isolated from Shunt lambs. Shunt PAECs were transduced with Ad-CA-Src (MOI = 10) for 48 h. Restoration of pp60^Src^ in Shunt PAECs increases ETC Complex I activity (**A**) and assembly (**B**). The bioenergetic profile is improved (**C**) such that basal respiration (**D**), the OCR for ATP synthesis (**E**), and the maximal (**F**) and reserve (**G**) respiratory capacity are significantly increased. Data are presented as means ± SEM. All experiments were performed with at least four biological replicates.

Finally, we explored if pp60^Src^ protein restoration in Shunt PAECs could recover ETC Complex I activity and Complex I assembly and restore mitochondrial bioenergetics. Restoration of pp60^Src^ improved ETC Complex I activity (Figure 8A) and increased ETC Complex I assembly (Figure 8B) in Shunt PAECs. OCR analysis using the Seahorse XFe24 extracellular flux analyzer in conjunction with the Mitostress assay revealed that bioenergetics were enhanced in CA-Src-expressing Shunt PAECs (Figure 8C) such that the basal respiration (Figure 8D), the O_2_ consumed for ATP production (Figure 8E), as well as the maximal (Figure 8F) and reserve (Figure 8G) respiratory capacity were all increased.

## 3. Discussion

pp60^Src^ is among the numerous abundant and highly functional kinases in vascular cells [46]. Evidence suggests that dysregulation/abnormal levels of pp60^Src^ lead to pathologic processes in vascular biology, including PH development [47,48,49,50,51]. Previously, we and others have reported irregular levels of pp60^Src^ in the monocrotaline-induced rat model of PH [43,44]. However, more information is needed regarding the role of pp60^Src^ in PH development. In vascular endothelial cells, pp60^Src^ mediates the tyrosine phosphorylation of numerous molecules involved in endothelial monolayer permeability [52]. Importantly, pp60^Src^ is also translocated to the mitochondria, phosphorylating resident proteins and modulating redox signaling [31,53]. The protein subunits of the respiratory complex I component can also be phosphorylated by pp60^Src^, which appears to be essential for preserving respiratory complex I function [54]. However, the physiological role and molecular targets of pp60^Src^ in the mitochondria still need to be fully understood. This study addressed this. Our study has three significant findings. First, mitochondrial pp60^Src^ levels are decreased in PAECs isolated from a lamb model of PH, affecting ETC Complex I assembly, activity, and mitochondrial bioenergetics. Second, we confirmed that the inhibition or knockdown of pp60^Src^ in PAECs is sufficient to attenuate ETC Complex I assembly, ETC Complex I activity, and mitochondrial bioenergetics. Third, restoring pp60^Src^ in Shunt PAECs reversed these changes and improved Complex I activity and assembly and mitochondrial bioenergetics. These fundamental biochemical findings significantly advance our understanding of the role of pp60^Src^ in mitochondrial regulation in PH.

Previously, we identified deficiencies in the activities of Complexes I, II, and III of the ETC in vascular cells isolated from a monocrotaline-induced rat model of PH [16]. Further, the defect in Complex I activity correlated with a loss in its assembly, although the assembly of Complexes II and III was maintained. In this study, we utilized a lamb PH model (Shunt) [55,56]. This model shares clinical and pathologic sequelae similar to children born with congenital heart defects that result in increased pulmonary blood flow [57]. We found that PAECs isolated from this Shunt model also showed mitochondrial dysfunction that correlated with deficiencies in Complexes I and II activities. However, the activity of Complex III was unaffected. Significantly, Complex I assembly was also attenuated in Shunt PAECs. Thus, these studies suggest that derangements in Complex I also impact the activities of other Complexes in the ETC [16]. Further, our data suggest that mitochondrial pp60^Src^ activity, particularly in the context of Complex I, plays a crucial role in regulating oxidative phosphorylation [37]. Our data support prior work showing that the Src family kinase inhibitor PP2 decreases Complex I assembly and activity and impacts mitochondrial function [58,59]. Although PP2 is a selective inhibitor for Src, it may also inhibit other Src family kinases [60] and induce the off-target inhibition of additional kinases [61]. To address this issue, we also employed a molecular approach utilizing an siRNA to reduce pp60^Src^ protein expression specifically. Using this approach, we confirmed that specifically targeting pp60^Src^ decreased Complex I assembly and activity and attenuated mitochondrial respiration. Together, these data suggest that decreases in mitochondrial pp60^Src^ may play a significant role in the mitochondrial dysfunction in PH due to its ability to regulate Complex I.

ETC Complex I, also called NADH-ubiquinone oxidoreductase, is the largest multi-subunit enzyme complex in the ETC. The function of ETC Complex I is to transfer electrons from the matrix NADH to ubiquinone. Complex I is composed of 45 proteins that are involved in the assembly and stabilization of the multimeric complex, the regulation of activity, and protection against reactive oxygen species [62,63]. Dysfunction of any of the components of the ETC could lead to energy deprivation, oxidative stress, and pathologic outcomes [64]. Protein conformation, stability, and activity can be regulated through different post-translational modifications, including phosphorylation, nitration, glutathionylation, and acetylation [65,66,67,68]. Mitochondrial protein phosphorylation can critically affect the properties of the resident proteins, including stability activity, and so regulate critical mitochondrial function [69]. This study found that pp60^Src^ inhibition by PP2 resulted in decreased pY levels in three mitochondrial Complex I proteins, which reduced ETC Complex I assembly and activity. Our results are supported by a prior study, which reported that mitochondrial complexes I and IV subunits are substrates for kinases, and the kinases-mediated phosphorylation led to a substantial increase in the activity of complexes I and IV. At the same time, dephosphorylation suppressed the activity of complexes I and IV [70]. The results from our study suggest that ETC Complex I activity partly depends on the pY PTMs in its subunits. This is supported by previous studies that demonstrated that the phosphorylation of ETC Complex I regulates both the activation and suppression of its activity [71,72]. Thus, it is likely that the magnitude of both phosphorylation and dephosphorylation in the regulation of mitochondrial function are controlled explicitly by kinases and phosphatases in the mitochondria [26,73,74,75,76]. Mitochondrial tyrosine phosphorylation is, thus, a central mechanism for regulating mitochondrial function. This is likely because the phosphorylation state of a protein influences many properties, including stability, enzymatic activity, and the ability to interact with binding partners. Since phosphorylation is rapidly reversible, it is an attractive signaling mechanism.

We identified three Complex I proteins as pp60^Src^ targets. The first, NDUFS1, encodes the NADH-ubiquinone oxidoreductase 75 kDa subunit. This is the largest subunit of ETC Complex I, accommodating three iron-sulfur clusters in the N-module, which binds and oxidizes NADH [77]. A previous study reported that mutations in NDUFS1 lead to metabolic reprogramming and disruption of the electron transfer [78]. Importantly, isolated fibroblast cells showed mitochondrial dysfunction and dysregulated metabolites affecting glycolytic activities. Thus, NDUSF1 is a critical regulator of ETC Complex I and bioenergetics. It has been previously reported that NDUFS1 is a target for Src kinase [31,79]. However, even with these critical new data, the physiological significance of the phosphorylation and dephosphorylation status of NDUFS1 remains unclear, and further research is warranted to investigate which pY PTMs in NDUFS1 are critical for ETC Complex I activity. We also identified NADH–ubiquinone oxidoreductase complex assembly factor 5 (NDUFAF5) as a substrate for pp60^Src^. NDUFAF5 encodes Arginine hydroxylase which catalyzes the hydroxylation of Arg^73^ in the NDUFS7 subunit and is essential for the assembly of ETC Complex I [80]. Previously, it has been reported that mutations in NDUFAF5 are associated with Leigh syndrome due to attenuated ETC Complex I assembly [81,82]. Although these mutations decrease ETC Complex I activity and assembly, little is known about the protein’s function and the disease’s mechanisms. The third complex I subunit phosphorylated by pp60^Src^ was NDUFV2, which encodes the 24-kD subunit of the mitochondrial NADH–ubiquinone oxidoreductase. Deficiency of this subunit causes hypertrophic cardiomyopathy and encephalopathy [83]. It has been reported that NDUFV2 is a target for pp60^Src^ in the mitochondria [84]. Notably, both NDUFS1 and NDUFV2 are subunits of the NADH dehydrogenase module (N module) responsible for the oxidation of NADH. Thus, it is likely that pp60^Src^-mediated phosphorylation of both NDUFS1 and NDUFV2 is critical for the assembly and stability of the N module. Further, a stabilized and functional N module can attach to the Q-module (electron transfer to ubiquinone) and P-module (proton pumping), forming the active mitochondrial respiratory Complex I. A fully assembled Complex I transfers electrons from NADH to Coenzyme Q and maintains the proton electrochemical gradient across the inner mitochondrial membrane [63]. Further, Complex I also provides stability to the multimeric complex, improving the activity of other ETC complexes and protecting against oxidation by reactive oxygen species. Complex I deficiency is the most known enzyme deficiency in patients with mitochondrial disorders. However, all complex I deficiencies have been associated with genetic defects with mutations reported for 14 core subunits [63]. Thus, further studies are required to improve our understanding of how PTM defects in respiratory complex subunits contribute to mitochondrial disorders. Additional work will also be required to identify THE pY site in the NDUFS1, NDUFAF5, and NDUFV2 I subunits to test the effects of loss of individual pY PTMs on ETC Complex I assembly, activity, and mitochondrial bioenergetics.

The phosphorylation and dephosphorylation of proteins are finely balanced in the cell and are known to regulate several protein–protein interactions. The dysregulation of phosphorylation and dephosphorylation events potentially leads to the pathogenesis of many disease processes, including PH [85,86]. However, limited knowledge is available about the phosphorylation and dephosphorylation status of mitochondrial proteins, including respiratory complex subunits and their involvement in the development of PH. We have previously shown that mitochondrial function is decreased in the PAECs isolated from our lamb model of PH [21]. Further, our studies demonstrate that mitochondrial dysfunction occurs secondary to disruptions in carnitine homeostasis and fatty acid oxidation in children born with complex congenital heart defects [87,88,89]. However, the potential contributors to the disruption of mitochondrial bioenergetics have not been adequately resolved. Interestingly, in this study, we observed decreased pp60^Src^ protein levels in the PH-PAECs. Further fluorescent microscopy investigation revealed decreased total pp60^Src^ levels and mitochondrially localized pp60^Src^ in PH-PAECs. Notably, the decrease in mitochondrial pp60^Src^ levels correlated with a reduction in the phosphorylation of the three mitochondrial Complex I subunits, impacting ETC Complex I activity, assembly, and bioenergetics. Restoring pp60^Src^ levels in Shunt PAECs improved Complex I activity, assembly, and mitochondrial bioenergetics. Together, our results establish the contribution of mitochondrial pp60^Src^ in regulating mitochondrial function in PH. It is also worth noting that pp60^Src^ lacks a mitochondrial localization signal and is dependent on single or multiple adaptor proteins for its translocation into mitochondria [31]. Thus, the expression of these adaptor proteins may also be disrupted in PH, which could also reduce mitochondrially localized pp60^Src^. This is an essential issue as prior work in more advanced models of PH has suggested that cytosolic pp60^Src^ levels are increased [43,44]. It is possible that reducing mitochondrial pp60^Src^ could induce metabolic reprogramming, which, like cancer, is associated with PH [90]. However, further studies will be required to delineate the potential differential effects of sub-cellular pools of pp60^Src^ on cell metabolism.

## 4. Materials and Methods

### 4.1. Antibodies, Reagents, and Chemicals

The antibodies against Src, phospho-Tyrosine (P-Tyr-100) were purchased from Cell Signaling Technologies (Danvers, MA, USA). The antibodies against NDUFS1, NDUFV2 and TOMM20 were purchased from Thermo Fisher (Waltham, MA, USA). The antibody against NDUFAF5 was purchased from Abcam (Waltham, MA, USA). The antibody against β-actin was purchased from Sigma (St. Louis, MO, USA). The secondary anti-mouse IgG (HRP-linked) and anti-rabbit IgG (HRP-linked) antibodies were purchased from Cell Signaling Technologies (Danvers, MA, USA). The Cy2 (green) AffiniPure donkey Anti-Rabbit IgG and the Cy3 (red) AffiniPure goat Anti-Mouse IgG secondary antibodies were purchased from Jackson ImmunoResearch (West Grove, PA, USA). PP2 was purchased from Sigma (St. Louis, MO, USA). All seahorse kits, reagents, and assay medium were purchased from Agilent Technology (Santa Clara, CA, USA). Unless specified, all other chemicals were purchased from Sigma (St. Louis, MO, USA) or Thermo Fisher (Waltham, MA, USA).

### 4.2. Cell Culture and Adenoviral Transduction of Pulmonary Arterial Endothelial Cells (PAECs)

As described previously [55,56], an 8.0 mm, ~2 mm length Gore-tex^®^ vascular graft was anastomosed between the ascending aorta and main pulmonary artery in anesthetized late gestation fetal lambs (137–141 days gestation; term = 145 days). Approximately four weeks after spontaneous delivery, lambs were sacrificed, and PAECs were isolated and cultured from three independent Shunt and three age-matched Control lambs following our previously published protocol [91,92]. For all the experiments, PAECs were used between passages 5 and 8. An adenoviral expression construct containing a constitutively active pp60^Src^ mutant (Ad-CA-Src) was constructed following a previously published protocol [93]. Shunt PAECs were transduced with/without adenovirus Ad-CA-Src (MOI = 10). Cells were allowed to grow for 48 h at 37 °C in an incubator with 5% CO_2_. In separate experiments, Control PAECs were treated with increasing concentrations of PP2 (0, 2.5, 5 and 10 µM) for 24 h.

### 4.3. Small Interfering RNA (siRNA) Treatment

Human PAECs (HPAECs) and the endothelial cell growth basal medium-2 were purchased from Lonza Bioscience (Allendale, NJ, USA). Scrambled siRNA, the pp60^Src^-specific siRNA, and transfection reagents were purchased from Dharmacon (Horizon Discovery Ltd., Cambridge, UK). The DharmaFECT siRNA transfection protocol was used to deliver the siRNAs. Cells were incubated at 37 °C in 5% CO_2_ for 48 h before protein analysis.

### 4.4. Mitochondria Isolation and Blue Native Polyacrylamide Gel Electrophoresis (BN-PAGE)

Mitochondria was isolated from PAECs using the Mitochondria Isolation Kit for Cultured Cells (Thermo Scientific, Waltham, MA, USA) using the manufacturer’s protocol. The blue native polyacrylamide gel electrophoresis method was carried out using the Invitrogen NativePAGE Novex Bis-Tris Gel System (Thermo Scientific, Waltham, MA, USA) and following previously described protocols [94]. Briefly, isolated mitochondria were resuspended in sample buffer containing NativePAGE sample buffer and 5% digitonin and incubated on ice for 20 min. Samples were centrifuged at 20,000× g for 10 min at 4 °C and mitochondrial protein concentration was determined. Coomassie G-250 sample additive (1 µL) was added to 10 µg of mitochondrial extract and samples were run on NativePAGE 3–12% gradient gel at 4 °C. Electrophoresis was set at 150 V for 30 min until the protein sample entered the stacking gel, followed by electrophoresis at 250 V for 150 min. The gels were stained with 0.1% Coomassie Brilliant Blue G 250 in a solution containing distilled water/methanol/acetic acid (50/40/10). Gels were subsequently transferred to the destaining solution containing distilled water/methanol/acetic acid (50/40/10). Blue Native PAGE gels were visualized and the respiratory complex band intensities were quantified using the Invitrogen iBright Imaging System (Thermo Scientific, Waltham, MA, USA).

### 4.5. Immunoprecipitation and Western Blot Analysis

The immunoprecipitation method was performed following the previously described protocol [95]. Briefly, cell lysates were mixed with 5 µg of respective antibodies and incubated overnight at 4 °C with mixing to form the immune complex. The antigen sample/antibody mixture was added to protein A/G agarose beads (Sigma, St. Louis, MO, USA) and incubated at room temperature (RT) for 1 h with mixing. Samples were washed with RIPA buffer and the eluted protein was mixed with SDS-PAGE sample buffer and heated at 95 °C for 5 min. the Western blot method was performed following the previously described protocol [96].

### 4.6. Phosphoprotein Enrichment and 2-Dimentional Electrophoresis

The Phosphoprotein Enrichment Kit (Thermo Scientific, Waltham, MA, USA) was used to enrich mitochondrial phosphorylated proteins derived from PAECs following the manufacturer’s described protocol. The two-dimensional polyacrylamide gel electrophoresis (2D-PAGE) method was performed following the previously described method [97]. Briefly, 7 cm, pH 3–10, immobilized pH gradient (IPG) strip (Bio-Rad, Hercules, CA, USA) were placed in contact with the protein samples in a rehydration buffer containing 2% IPG buffer (pH 3 to 10), 2% dithiothreitol (DTT), 7 M urea, 2 M thiourea, 4% (wt/vol) CHAPS, and 0.5% bromophenol blue. The strips were subjected to first-dimension separation by using the PROTEAN i12 IEF system (Bio-Rad, Hercules, CA, USA), with the following protocol: temperature of 20 °C; current of 50 A per strip; and 300 V (step) for 30 min, 1000 V (gradient) for 30 min, 5000 V (gradient) for 85 min, and 5000 V (step) for 25 min. The strips were incubated in equilibration buffer (6 M urea, 50 mM Tris [pH 8.8], 2% SDS, 30% glycerol, 0.5% bromophenol blue) with 1% DTT for 10 min, followed by incubation in equilibration buffer with 2.5% (wt/vol) iodoacetamide for 10 min. The equilibrated strips were electrophoresed on NuPAGE Novex 4 to 12% IPG Well Bis-Tris gels (Invitrogen, Carlsbad, CA, USA). The gels were incubated overnight in the Coomassie Blue staining solution. Gels were transferred to the destaining solution containing distilled water. The gels were visualized through scanning for Coomassie staining using iBright Imaging Systems (Invitrogen, Carlsbad, CA, USA).

### 4.7. DE Gel Analysis for Protein Expression Profiling

The Melanie software (Cytiva, Wilmington, DE, USA) was used to visualize, match, detect, quantitate, and analyze protein spots on 2D-PAGE images following the manufacturer’s protocol [98,99].

### 4.8. In-Gel Protein Digestion

In-gel digestion was performed for proteins separated by 2D electrophoresis. After detecting Coomassie-stained proteins in polyacrylamide gels, protein spots from the 2D gel were excised, destained with 50 mM ammonium bicarbonate (pH 8) (Sigma, St. Louis, MO, USA) and incubated with 20 mM DTT for 1 h at 60 °C. Next, 40 mM iodoacetamide was added to the reduced protein samples, and the reaction mixture was incubated for 30 min, at RT protected from light. In-gel trypsin/chymotrypsin digestion (Thermo Fisher, Waltham, MA, USA) was performed overnight at 37 °C. Extracted peptide samples were purified using C18 tips (Thermo Fisher, Waltham, MA, USA) and resuspended in 0.1% Formic Acid (Thermo Fisher, Waltham, MA, USA).

### 4.9. Mass Spectrometry

Liquid chromatography–tandem mass spectrometry (LC-MS/MS) was carried out using a nanoElute nanoflow LC system coupled to the timsTOF fleX MALDI-2 mass spectrometer (Bruker Daltonics, Billerica, MA, USA), following our previous study protocol [100].

### 4.10. Fluorescent Microscopy

PAECs were plated and grown on coverslips in a 24-well plate. Upon reaching confluency, cells were fixed with 4% formaldehyde. Cells were probed overnight at 4 °C with the Src Rabbit and TOMM20 Mouse primary antibody in a 1:500 dilution in 2.5% BSA in PBS. The next day, Cy2 (green) AffiniPure donkey Anti-Rabbit IgG and the Cy3 (red) AffiniPure goat Anti-Mouse IgG secondary antibodies were added to the coverslips in a 1:1000 dilution in 2.5% BSA in PBS for 90 min at RT. The nucleus was stained with DAPI (4′,6-diamidino-2-phenylindole, dihydrochloride) (Thermo Fisher, Waltham, MA, USA) and coverslips were mounted using ProLong Antifade Mountant (Thermo Fisher, Waltham, MA, USA). Low-noise and high-sensitivity fluorescence images were then captured using a KEYENCE BZ-X800 fluorescence microscope. For microcopy analysis, 5 replicates were performed per cell line. For each replicate, 2 images of ~50 cells were obtained and analyzed. All images were taken using z-stacks with a 0.2 µm pitch at the following exposure times: green (Cy2): 1/5 seconds, red (Cy3): 1/3 s, blue: 1/250 s. After capture, the Z-stack was merged into a full-focus image, and background fluorescence was subtracted from the image. Haze reduction was performed with a reduction of 0.3. Haze-reduced images were used as representative images for clarity, analysis was performed on images without the haze reduction. For histogram analysis, images without haze reduction were analyzed. ImageJ Fiji v1.54p was used to obtain the mean fluorescence intensity and standard deviation of red, green, and blue channels. To quantify colocalization, Pearson’s correlation coefficients and the Manders’ Split Coefficient were obtained using Just Another Colocalization Plugin (JACoP) in ImageJ Fiji with appropriate thresholds to count all significant intensities.

### 4.11. Measurement of Oxygen Consumption Rate

The XFe24 Analyzer (Agilent Technologies, Santa Clara, CA, USA) and XF Cell Mito Stress Test Kit (103015-100; Agilent Technologies, Santa Clara, CA, USA) were used for the mitochondrial bioenergetic analyses. The oxygen consumption rate was measured following a previously published protocol [96].

### 4.12. Functional Analysis of Measurement of Electron Transport Chain (ETC) Complex I

The XFe24 Analyzer was used to measure Complex I activity as previously described [16]. The activity of ETC Complex I was examined utilizing mix/wait/measure times of 0.5 min/0.5 min/2 min with no equilibration step and two measurements per step.

### 4.13. Statistical Analysis

Statistical analysis for this project was performed using GraphPad Prism version 4.01. The mean ± SEM was calculated for all samples and significance was determined using the unpaired *t*-test. A statistically significant test result *p* < 0.05 was accepted.

## 5. Conclusions

Our data show that mitochondrially localized pp60^Src^ is critical in regulating ETC Complex I assembly, activity, and mitochondrial bioenergetics. Further, the pY levels of NDUFS1, NDUFAF5, and NDUFV2 are decreased in PAECs isolated from a lamb model of PH, suggesting they are essential for disease pathogenesis. However, further studies will be required to understand the molecular mechanisms and set points involved in mitochondrial pp60^Src^-mediated phosphorylation and how disrupting these leads to the development of cardiopulmonary diseases. Further research is also warranted to identify, map, and quantify untraced PTMs in the mitochondrial respiratory complex subunits and investigate their contribution to mitochondrial dysfunction and the development of PH.

## Data Availability

Original data are available on request to the corresponding author.

## References

[1] Vakifahmetoglu-Norberg H., Ouchida A.T., Norberg E. (2017). The role of mitochondria in metabolism and cell death. Biochem. Biophys. Res. Commun..

[2] Federico M., De la Fuente S., Palomeque J., Sheu S.-S. (2021). The role of mitochondria in metabolic disease: A special emphasis on heart dysfunction. J. Physiol..

[3] Kwak S.H., Park K.S., Lee K.-U., Lee H.K. (2010). Mitochondrial metabolism and diabetes. J. Diabetes Investig..

[4] Grasso D., Zampieri L.X., Capelôa T., Van de Velde J.A., Sonveaux P. (2020). Mitochondria in cancer. Cell Stress.

[5] Missiroli S., Perrone M., Genovese I., Pinton P., Giorgi C. (2020). Cancer metabolism and mitochondria: Finding novel mechanisms to fight tumours. eBioMedicine.

[6] Poznyak A.V., Ivanova E.A., Sobenin I.A., Yet S.-F., Orekhov A.N. (2020). The Role of Mitochondria in Cardiovascular Diseases. Biology.

[7] Chistiakov D.A., Shkurat T.P., Melnichenko A.A., Grechko A.V., Orekhov A.N. (2018). The role of mitochondrial dysfunction in cardiovascular disease: A brief review. Ann. Med..

[8] Liang S., Yegambaram M., Wang T., Wang J., Black S.M., Tang H. (2022). Mitochondrial Metabolism, Redox, and Calcium Homeostasis in Pulmonary Arterial Hypertension. Biomedicines.

[9] Hroudová J., Singh N., Fišar Z. (2014). Mitochondrial Dysfunctions in Neurodegenerative Diseases: Relevance to Alzheimer’s Disease. BioMed Res. Int..

[10] Wang W., Zhao F., Ma X., Perry G., Zhu X. (2020). Mitochondria dysfunction in the pathogenesis of Alzheimer’s disease: Recent advances. Mol. Neurodegener..

[11] Budzinska M., Zimna A., Kurpisz M. (2021). The role of mitochondria in Duchenne muscular dystrophy. J. Physiol. Pharmacol..

[12] Pokharel M.D., Marciano D.P., Fu P., Franco M.C., Unwalla H., Tieu K., Fineman J.R., Wang T., Black S.M. (2023). Metabolic reprogramming, oxidative stress, and pulmonary hypertension. Redox Biol..

[13] Zhang W., Liu B., Wang Y., Zhang H., He L., Wang P., Dong M. (2022). Mitochondrial dysfunction in pulmonary arterial hypertension. Front. Physiol..

[14] Suliman H.B., Nozik-Grayck E. (2019). Mitochondrial Dysfunction: Metabolic Drivers of Pulmonary Hypertension. Antioxid. Redox Signal..

[15] Marshall J.D., Bazan I., Zhang Y., Fares W.H., Lee P.J. (2018). Mitochondrial dysfunction and pulmonary hypertension: Cause, effect, or both. Am. J. Physiol. Lung Cell. Mol. Physiol..

[16] Rafikov R., Sun X., Rafikova O., Louise Meadows M., Desai A.A., Khalpey Z., Yuan J.X., Fineman J.R., Black S.M. (2015). Complex I dysfunction underlies the glycolytic switch in pulmonary hypertensive smooth muscle cells. Redox Biol..

[17] Ma W., Zhang P., Vang A., Zimmer A., Huck S., Nicely P., Wang E., Mancini T.J., Owusu-Sarfo J., Cavarsan C.F. (2024). Reduction in activity and abundance of mitochondrial electron transport chain supercomplexes in pulmonary hypertension-induced right ventricular dysfunction. bioRxiv.

[18] Mooers E.A., Johnson H.M., Michalkiewicz T., Rana U., Joshi C., Afolayan A.J., Teng R.J., Konduri G.G. (2024). Aberrant PGC-1alpha signaling in a lamb model of persistent pulmonary hypertension of the newborn. Pediatr. Res..

[19] Redout E.M., Wagner M.J., Zuidwijk M.J., Boer C., Musters R.J., van Hardeveld C., Paulus W.J., Simonides W.S. (2007). Right-ventricular failure is associated with increased mitochondrial complex II activity and production of reactive oxygen species. Cardiovasc. Res..

[20] Yang Z., Zhuan B., Yan Y., Jiang S., Wang T. (2016). Roles of different mitochondrial electron transport chain complexes in hypoxia-induced pulmonary vasoconstriction. Cell Biol. Int..

[21] Sun X., Lu Q., Yegambaram M., Kumar S., Qu N., Srivastava A., Wang T., Fineman J.R., Black S.M. (2020). TGF-beta1 attenuates mitochondrial bioenergetics in pulmonary arterial endothelial cells via the disruption of carnitine homeostasis. Redox Biol..

[22] Ardito F., Giuliani M., Perrone D., Troiano G., Lo Muzio L. (2017). The crucial role of protein phosphorylation in cell signaling and its use as targeted therapy (Review). Int. J. Mol. Med..

[23] Zhang W., Liu H.T. (2002). MAPK signal pathways in the regulation of cell proliferation in mammalian cells. Cell Res..

[24] Niemi N.M., MacKeigan J.P. (2013). Mitochondrial phosphorylation in apoptosis: Flipping the death switch. Antioxid. Redox Signal..

[25] Horbinski C., Chu C.T. (2005). Kinase signaling cascades in the mitochondrion: A matter of life or death. Free Radic. Biol. Med..

[26] Pagliarini D.J., Dixon J.E. (2006). Mitochondrial modulation: Reversible phosphorylation takes center stage?. Trends Biochem. Sci..

[27] McBride H.M., Neuspiel M., Wasiak S. (2006). Mitochondria: More than just a powerhouse. Curr. Biol..

[28] Koc E.C., Koc H. (2012). Regulation of mammalian mitochondrial translation by post-translational modifications. Biochim. Biophys. Acta (BBA)-Gene Regul. Mech..

[29] Hofer A., Wenz T. (2014). Post-translational modification of mitochondria as a novel mode of regulation. Exp. Gerontol..

[30] Tibaldi E., Brunati A.M., Massimino M.L., Stringaro A., Colone M., Agostinelli E., Arancia G., Toninello A. (2008). Src-Tyrosine kinases are major agents in mitochondrial tyrosine phosphorylation. J. Cell. Biochem..

[31] Hebert-Chatelain E. (2013). Src kinases are important regulators of mitochondrial functions. Int. J. Biochem. Cell Biol..

[32] Yeatman T.J. (2004). A renaissance for SRC. Nat. Rev. Cancer.

[33] Ishizawar R., Parsons S.J. (2004). c-Src and cooperating partners in human cancer. Cancer Cell.

[34] Demory M.L., Boerner J.L., Davidson R., Faust W., Miyake T., Lee I., Hüttemann M., Douglas R., Haddad G., Parsons S.J. (2009). Epidermal growth factor receptor translocation to the mitochondria: Regulation and effect. J. Biol. Chem..

[35] Bard F., Patel U., Levy J.B., Jurdic P., Horne W.C., Baron R. (2002). Molecular complexes that contain both c-Cbl and c-Src associate with Golgi membranes. Eur. J. Cell Biol..

[36] Kaplan K.B., Swedlow J.R., Varmus H.E., Morgan D.O. (1992). Association of p60c-src with endosomal membranes in mammalian fibroblasts. J. Cell Biol..

[37] Ge H., Zhao M., Lee S., Xu Z. (2015). Mitochondrial Src tyrosine kinase plays a role in the cardioprotective effect of ischemic preconditioning by modulating complex I activity and mitochondrial ROS generation. Free Radic. Res..

[38] Johnson Kameny R., Datar S.A., Boehme J.B., Morris C., Zhu T., Goudy B.D., Johnson E.G., Galambos C., Raff G.W., Sun X. (2019). Ovine Models of Congenital Heart Disease and the Consequences of Hemodynamic Alterations for Pulmonary Artery Remodeling. Am. J. Respir. Cell Mol. Biol..

[39] Xu W., Erzurum S.C. (2011). Endothelial cell energy metabolism, proliferation, and apoptosis in pulmonary hypertension. Compr. Physiol..

[40] Sun X., Sharma S., Fratz S., Kumar S., Rafikov R., Aggarwal S., Rafikova O., Lu Q., Burns T., Dasarathy S. (2013). Disruption of endothelial cell mitochondrial bioenergetics in lambs with increased pulmonary blood flow. Antioxid. Redox Signal..

[41] Shi Y., Liu J., Zhang R., Zhang M., Cui H., Wang L., Cui Y., Wang W., Sun Y., Wang C. (2023). Targeting Endothelial ENO1 (Alpha-Enolase) -PI3K-Akt-mTOR Axis Alleviates Hypoxic Pulmonary Hypertension. Hypertension.

[42] Xu W., Koeck T., Lara A.R., Neumann D., DiFilippo F.P., Koo M., Janocha A.J., Masri F.A., Arroliga A.C., Jennings C. (2007). Alterations of cellular bioenergetics in pulmonary artery endothelial cells. Proc. Natl. Acad. Sci. USA.

[43] Rafikova O., Rafikov R., Kangath A., Qu N., Aggarwal S., Sharma S., Desai J., Fields T., Ludewig B., Yuan J.X.Y. (2016). Redox regulation of epidermal growth factor receptor signaling during the development of pulmonary hypertension. Free. Radic. Biol. Med..

[44] Paulin R., Meloche J., Courboulin A., Lambert C., Haromy A., Courchesne A., Bonnet P., Provencher S., Michelakis E.D., Bonnet S. (2014). Targeting cell motility in pulmonary arterial hypertension. Eur. Respir. J..

[45] Dai X., Wang L.J., Wu J., Shi Y.X., Li G.P., Yang X.Q. (2018). Src kinase inhibitor PP2 regulates the biological characteristics of A549 cells via the PI3K/Akt signaling pathway. Oncol. Lett..

[46] Oda Y., Renaux B., Bjorge J., Saifeddine M., Fujita D.J., Hollenberg M.D. (1999). cSrc is a major cytosolic tyrosine kinase in vascular tissue. Can. J. Physiol. Pharmacol..

[47] MacKay C.E., Knock G.A. (2015). Control of vascular smooth muscle function by Src-family kinases and reactive oxygen species in health and disease. J. Physiol..

[48] Chou M.T., Wang J., Fujita D.J. (2002). Src Kinase becomes preferentially associated with the VEGFR, KDR/Flk-1, following VEGF stimulation of vascular endothelial cells. BMC Biochem..

[49] Wallez Y., Cand F., Cruzalegui F., Wernstedt C., Souchelnytskyi S., Vilgrain I., Huber P. (2007). Src kinase phosphorylates vascular endothelial-cadherin in response to vascular endothelial growth factor: Identification of tyrosine 685 as the unique target site. Oncogene.

[50] Duval M., Bœuf F.L., Huot J., Gratton J.-P. (2007). Src-mediated Phosphorylation of Hsp90 in Response to Vascular Endothelial Growth Factor (VEGF) Is Required for VEGF Receptor-2 Signaling to Endothelial NO Synthase. Mol. Biol. Cell.

[51] Pullamsetti S.S., Berghausen E.M., Dabral S., Tretyn A., Butrous E., Savai R., Butrous G., Dahal B.K., Brandes R.P., Ghofrani H.A. (2012). Role of Src tyrosine kinases in experimental pulmonary hypertension. Arterioscler. Thromb. Vasc. Biol..

[52] Hu G., Place A.T., Minshall R.D. (2008). Regulation of endothelial permeability by Src kinase signaling: Vascular leakage versus transcellular transport of drugs and macromolecules. Chem. Biol. Interact..

[53] Miyazaki T., Neff L., Tanaka S., Horne W.C., Baron R. (2003). Regulation of cytochrome c oxidase activity by c-Src in osteoclasts. J. Cell Biol..

[54] Hebert-Chatelain E., Jose C., Gutierrez Cortes N., Dupuy J.W., Rocher C., Dachary-Prigent J., Letellier T. (2012). Preservation of NADH ubiquinone-oxidoreductase activity by Src kinase-mediated phosphorylation of NDUFB10. Biochim. Biophys. Acta (BBA) Bioenerg..

[55] Oishi P.E., Sharma S., Datar S.A., Kumar S., Aggarwal S., Lu Q., Raff G., Azakie A., Hsu J.H., Sajti E. (2013). Rosiglitazone preserves pulmonary vascular function in lambs with increased pulmonary blood flow. Pediatr. Res..

[56] Oishi P.E., Wiseman D.A., Sharma S., Kumar S., Hou Y., Datar S.A., Azakie A., Johengen M.J., Harmon C., Fratz S. (2008). Progressive dysfunction of nitric oxide synthase in a lamb model of chronically increased pulmonary blood flow: A role for oxidative stress. Am. J. Physiol. Lung Cell. Mol. Physiol..

[57] Black S.M., Fineman J.R., Johengen M., Bristow J., Soifer S.J. (1996). Increased Pulmonary Blood Flow Alters the Molecular Regulation of Vascular Reactivity in the Lamb. • 122. Pediatr. Res..

[58] Seltana A., Guezguez A., Lepage M., Basora N., Beaulieu J.-F. (2013). Src family kinase inhibitor PP2 accelerates differentiation in human intestinal epithelial cells. Biochem. Biophys. Res. Commun..

[59] Alcalá S., Mayoral-Varo V., Ruiz-Cañas L., López-Gil J.C., Heeschen C., Martín-Pérez J., Sainz B. (2020). Targeting SRC Kinase Signaling in Pancreatic Cancer Stem Cells. Int. J. Mol. Sci..

[60] Bain J., Plater L., Elliott M., Shpiro N., Hastie C.J., Mclauchlan H., Klevernic I., Arthur J.S.C., Alessi D.R., Cohen P. (2007). The selectivity of protein kinase inhibitors: A further update. Biochem. J..

[61] Brandvold K.R., Steffey M.E., Fox C.C., Soellner M.B. (2012). Development of a highly selective c-Src kinase inhibitor. ACS Chem. Biol..

[62] McKenzie M., Ryan M.T. (2010). Assembly factors of human mitochondrial complex I and their defects in disease. IUBMB Life.

[63] Mimaki M., Wang X., McKenzie M., Thorburn D.R., Ryan M.T. (2012). Understanding mitochondrial complex I assembly in health and disease. Biochim. Biophys. Acta (BBA) Bioenerg..

[64] Sharma L.K., Lu J., Bai Y. (2009). Mitochondrial respiratory complex I: Structure, function and implication in human diseases. Curr. Med. Chem..

[65] Cassina A.M., Hodara R., Souza J.M., Thomson L., Castro L., Ischiropoulos H., Freeman B.A., Radi R. (2000). Cytochrome c nitration by peroxynitrite. J. Biol. Chem..

[66] Wang T., Yegambaram M., Gross C., Sun X., Lu Q., Wang H., Wu X., Kangath A., Tang H., Aggarwal S. (2021). RAC1 nitration at Y32 IS involved in the endothelial barrier disruption associated with lipopolysaccharide-mediated acute lung injury. Redox Biol..

[67] Beer S.M., Taylor E.R., Brown S.E., Dahm C.C., Costa N.J., Runswick M.J., Murphy M.P. (2004). Glutaredoxin 2 catalyzes the reversible oxidation and glutathionylation of mitochondrial membrane thiol proteins: Implications for mitochondrial redox regulation and antioxidant DEFENSE. J. Biol. Chem..

[68] Guan K.L., Xiong Y. (2011). Regulation of intermediary metabolism by protein acetylation. Trends Biochem. Sci..

[69] Cesaro L., Salvi M. (2010). Mitochondrial tyrosine phosphoproteome: New insights from an up-to-date analysis. Biofactors.

[70] Falfushynska H.I., Sokolov E., Piontkivska H., Sokolova I.M. (2020). The Role of Reversible Protein Phosphorylation in Regulation of the Mitochondrial Electron Transport System During Hypoxia and Reoxygenation Stress in Marine Bivalves. Front. Mar. Sci..

[71] Lucero M., Suarez A.E., Chambers J.W. (2019). Phosphoregulation on mitochondria: Integration of cell and organelle responses. CNS Neurosci. Ther..

[72] Mathers K.E., Staples J.F. (2019). Differential posttranslational modification of mitochondrial enzymes corresponds with metabolic suppression during hibernation. Am. J. Physiol. Regul. Integr. Comp. Physiol..

[73] Salvi M., Brunati A.M., Bordin L., La Rocca N., Clari G., Toninello A. (2002). Characterization and location of Src-dependent tyrosine phosphorylation in rat brain mitochondria. Biochim. Biophys. Acta (BBA) Mol. Cell Res..

[74] Salvi M., Stringaro A., Brunati A.M., Agostinelli E., Arancia G., Clari G., Toninello A. (2004). Tyrosine phosphatase activity in mitochondria: Presence of Shp-2 phosphatase in mitochondria. Cell. Mol. Life Sci..

[75] Salvi M., Brunati A.M., Toninello A. (2005). Tyrosine phosphorylation in mitochondria: A new frontier in mitochondrial signaling. Free Radic. Biol. Med..

[76] Pagliarini D.J., Wiley S.E., Kimple M.E., Dixon J.R., Kelly P., Worby C.A., Casey P.J., Dixon J.E. (2005). Involvement of a mitochondrial phosphatase in the regulation of ATP production and insulin secretion in pancreatic beta cells. Mol. Cell.

[77] Brink J., Hovmöller S., Ragan C.I., Cleeter M.W.J., Boekema E.J., van Bruggen E.F.J. (1987). The structure of NADH:ubiquinone oxidoreductase from beef-heart mitochondria. Eur. J. Biochem..

[78] Ni Y., Hagras M.A., Konstantopoulou V., Mayr J.A., Stuchebrukhov A.A., Meierhofer D. (2019). Mutations in NDUFS1 Cause Metabolic Reprogramming and Disruption of the Electron Transfer. Cells.

[79] Hébert Chatelain E., Dupuy J.W., Letellier T., Dachary-Prigent J. (2011). Functional impact of PTP1B-mediated Src regulation on oxidative phosphorylation in rat brain mitochondria. Cell. Mol. Life Sci..

[80] Rhein V.F., Carroll J., Ding S., Fearnley I.M., Walker J.E. (2016). NDUFAF5 Hydroxylates NDUFS7 at an Early Stage in the Assembly of Human Complex, I. J. Biol. Chem..

[81] Sugiana C., Pagliarini D.J., McKenzie M., Kirby D.M., Salemi R., Abu-Amero K.K., Dahl H.H., Hutchison W.M., Vascotto K.A., Smith S.M. (2008). Mutation of C20orf7 disrupts complex I assembly and causes lethal neonatal mitochondrial disease. Am. J. Hum. Genet..

[82] Gerards M., Sluiter W., van den Bosch B.J., de Wit L.E., Calis C.M., Frentzen M., Akbari H., Schoonderwoerd K., Scholte H.R., Jongbloed R.J. (2010). Defective complex I assembly due to C20orf7 mutations as a new cause of Leigh syndrome. J. Med. Genet..

[83] Liu H.-Y., Liao P.-C., Chuang K.-T., Kao M.-C. (2011). Mitochondrial targeting of human NADH dehydrogenase (ubiquinone) flavoprotein 2 (NDUFV2) and its association with early-onset hypertrophic cardiomyopathy and encephalopathy. J. Biomed. Sci..

[84] Ogura M., Yamaki J., Homma M.K., Homma Y. (2012). Mitochondrial c-Src regulates cell survival through phosphorylation of respiratory chain components. Biochem. J..

[85] Cohen P. (2001). The role of protein phosphorylation in human health and disease. The Sir Hans Krebs Medal Lecture. Eur. J. Biochem..

[86] Weatherald J., Chaumais M.C., Savale L., Jaïs X., Seferian A., Canuet M., Bouvaist H., Magro P., Bergeron A., Guignabert C. (2017). Long-term outcomes of dasatinib-induced pulmonary arterial hypertension: A population-based study. Eur. Respir. J..

[87] Sharma S., Sud N., Wiseman D.A., Carter A.L., Kumar S., Hou Y., Rau T., Wilham J., Harmon C., Oishi P. (2008). Altered carnitine homeostasis is associated with decreased mitochondrial function and altered nitric oxide signaling in lambs with pulmonary hypertension. Am. J. Physiol.-Lung Cell. Mol. Physiol..

[88] Sharma S., Sun X., Kumar S., Rafikov R., Aramburo A., Kalkan G., Tian J., Rehmani I., Kallarackal S., Fineman J.R. (2012). Preserving mitochondrial function prevents the proteasomal degradation of GTP cyclohydrolase I. Free Radic. Biol. Med..

[89] Black S.M., Field-Ridley A., Sharma S., Kumar S., Keller R.L., Kameny R., Maltepe E., Datar S.A., Fineman J.R. (2017). Altered Carnitine Homeostasis in Children With Increased Pulmonary Blood Flow Due to Ventricular Septal Defects. Pediatr. Crit. Care Med..

[90] Liu X., Zhang L., Zhang W. (2022). Metabolic reprogramming: A novel metabolic model for pulmonary hypertension. Front. Cardiovasc. Med..

[91] Kelly L.K., Wedgwood S., Steinhorn R.H., Black S.M. (2004). Nitric oxide decreases endothelin-1 secretion through the activation of soluble guanylate cyclase. Am. J. Physiol. Lung Cell. Mol. Physiol..

[92] Wedgwood S., Mitchell C.J., Fineman J.R., Black S.M. (2003). Developmental differences in the shear stress-induced expression of endothelial NO synthase: Changing role of AP-1. Am. J. Physiol. Lung Cell. Mol. Physiol..

[93] Barabutis N., Handa V., Dimitropoulou C., Rafikov R., Snead C., Kumar S., Joshi A., Thangjam G., Fulton D., Black S.M. (2013). LPS induces pp60c-src-mediated tyrosine phosphorylation of Hsp90 in lung vascular endothelial cells and mouse lung. Am. J. Physiol. Lung Cell. Mol. Physiol..

[94] Van Coster R., Smet J., George E., De Meirleir L., Seneca S., Van Hove J., Sebire G., Verhelst H., De Bleecker J., Van Vlem B. (2001). Blue Native Polyacrylamide Gel Electrophoresis: A Powerful Tool in Diagnosis of Oxidative Phosphorylation Defects. Pediatr. Res..

[95] Sud N., Sharma S., Wiseman D.A., Harmon C., Kumar S., Venema R.C., Fineman J.R., Black S.M. (2007). Nitric oxide and superoxide generation from endothelial NOS: Modulation by HSP90. Am. J. Physiol. Lung Cell. Mol. Physiol..

[96] Yegambaram M., Sun X., Flores A.G., Lu Q., Soto J., Richards J., Aggarwal S., Wang T., Gu H., Fineman J.R. (2023). Novel Relationship between Mitofusin 2-Mediated Mitochondrial Hyperfusion, Metabolic Remodeling, and Glycolysis in Pulmonary Arterial Endothelial Cells. Int. J. Mol. Sci..

[97] Manivannan B., Rawson P., Jordan T.W., Secor W.E., La Flamme A.C. (2010). Differential patterns of liver proteins in experimental murine hepatosplenic schistosomiasis. Infect. Immun..

[98] Appel R.D., Palagi P.M., Walther D., Vargas J.R., Sanchez J.-C., Ravier F., Pasquali C., Hochstrasser D.F. (1997). Melanie II—A third-generation software package for analysis of two-dimensional electrophoresis images: I. Features and user interface. Electrophoresis.

[99] Appel R.D., Vargas J.R., Palagi P.M., Walther D., Hochstrasser D.F. (1997). Melanie II—A third-generation software package for analysis of two-dimensional electrophoresis images: II. Algorithms. Electrophoresis.

[100] Yegambaram M., Kumar S., Wu X., Lu Q., Sun X., Garcia Flores A., Meadows M.L., Barman S., Fulton D., Wang T. (2023). Endothelin-1 acutely increases nitric oxide production via the calcineurin mediated dephosphorylation of Caveolin-1. Nitric Oxide.

